# The Results of Operations for Cancer of the Breast

**Published:** 1908-08-29

**Authors:** T. Crisp English

**Affiliations:** Senior Assistant Surgeon to St. George's Hospital and to the Grosvenor Hospital for Women; Surgeon to the Hospital of St. John and St. Elizabeth.


### Hospital Clinics.

### THE RESULTS OF OPERATIONS FOR CANCER OF THE BREAST.

By T. CRISP ENGLISH, B.S., F.R.C.S., Senior Assistant Surgeon to St. George's Hospital and to the Grosvenor Hospital for Women; Surgeon to the Hospital of St. John and St. Elizabeth.

(Abstract of a paper read before the St. George's Hospital Hunterian Society.)

We still look in vain for a specific cure for cancer; the question of a cancer cure has during the past few years attracted considerable attention both in the medical profession and amongst the general public. Yet, so far, all attempts to discover the cause of, or a cure for, cancer have failed, and thus it comes about that the public belief in the hopelessness of cancer is confirmed, and the successes of surgery are overshadowed.

As a matter of fact, the proportion of cures after operations for cancer is steadily increasing, and nowhere is this steady progress so well shown as in the surgery of the breast. This result is due firstly to improvements in the methods of operating, and secondly, to the fact that patients are submitted to operation at a much earlier stage than was formerly the case.

The modern operation is based upon a better knowledge of the pathology of mammary cancer, and with it I believe that 50 per cent. of the cases may be expected to remain permanently cured; the more this is realised by the profession and the public, the earlier will patients submit to operation, and the higher will be the percentage of cures.

Although it is probable that operations for cancer of the breast will become still more thorough and extensive in the future, our efforts at the present time must be devoted mainly to getting the cases sooner, and to teaching the very earliest signs of the disease. The modern text-book description of mammary carcinoma needs revising: it teaches the symptoms and signs of the fully-established disease, but tells very little of the diagnosis of the disease in the earliest stage, when the prospects of cure are good. Almost all surgical papers on malignant disease urge that cases should be sent earlier for operation. This, of course, is quite right, but I think that practitioners are scarcely fairly treated, in that they get little or no credit for the fact that they send the cases infinitely earlier than formerly. Much of the improvement in the results of operations is due to the fact that the medical practitioners recognise the cases earlier, and so the surgeon gets the cases in much earlier stages.

### The Modern Operation.

Before discussing results it is necessary to refer briefly to the extent of the modern operation. The following structures are removed in one continuous mass: (1) a wide area of skin, having its centre over the centre of the growth; (2) the whole of the breast; (3) the lymphatic area and the fascia from the epigastrium to the clavicle, and from the sternum to the posterior axillary line, including the axillary fascia and lymphatics, as far as the termination of the subclavian vessels; (4) the whole of the pectoral muscles, with the exception of the upper fibres of the greater pectoral.

Removal of the pectoral muscles is an essential part of the operation. Unless this is done, it is impossible to make a clean dissection of the axilla right up to its apex, and upon the thoroughness of this dissection depends largely the ultimate result of the operation. There are many who strongly oppose routine removal of the muscles, saying that it is unjustifiable and unnecessary; but those who have practised it know that the advantages gained are enormous. It is true that it adds somewhat to the severity of the operation; but this is a minor matter compared with the increased prospect of cure and considering the terrible nature of the disease with which we are dealing.

The operation is therefore a very extensive one, and if conscientiously carried out, a very thorough one. Its usual duration is fifty to sixty minutes. A variable degree of shock follows in a minority of the cases, but this is soon recovered from; the patient is encouraged to move her arm after the third day, and usually is able to get up at the end of the week. In the greater number of the cases, there is subsequently little or no impairment of the movements of the arm and shoulder.

### Results of Thirty-two Operations.

Now as to the ultimate results of these operations. It is obvious that deductions from statistics must be made with great care, for the practice of individual surgeons varies greatly. Some surgeons perform the full operation in every case in which there is any hope of eradicating the disease; others reserve the full operation for advanced cases; again some operate upon very advanced cases which others would reject as hopeless. It is only possible to form a reliable opinion of the value of an operation of this kind by following up the patients from year to year, and I propose now to refer shortly to what has happened to the cases of mammary cancer upon which I have operated. My own cases are as yet small in number, and most have been watched for comparatively short periods only. I will, however, give the results for what they are worth. All the patients on whom the radical operation has been performed have been communicated with at least once in every six months since their operations; and in each case the diagnosis of malignant disease was confirmed microscopically.

There are ten cases in which operation was performed three or more years ago; of these eight are alive and well; one patient who had a large inflamed carcinoma died of pulmonary deposits without local recurrence six months after operation, and it is practically certain that these deposits were present before operation; one patient developed recurrence behind the clavicle three and a half years after operation; I made an attempt to remove this, but found that the disease extended deeply into the neck and towards the mediastinum; of the eight patients who have remained free from recurrence, one had an ulcerating growth in the upper and outer quadrant of the breast, two had extensive involvement of the axillary glands, and two had growths which were reported to be dual carcinomata.

There are seventeen patients upon whom I operated from one to three years ago. Three of these have died of internal deposits without local recurrence: (1) deposits in lungs, pleuræ and mediastinal glands, died thirteen months after operation; (2) multiple deposits in chest and abdomen, died one year after operation; (3) left breast removed one year previously by another surgeon for a malignant endothelioma; right breast removed in November 1906; died fifteen months later without local recurrence. A fourth patient died twenty months after operation apparently from heart failure; the doctor wrote saying that he could find no evidence of recurrence locally or elsewhere; no post-mortem was performed. Another patient aged forty-three developed two nodules in the scar and a gland above the clavicle, these were removed, and she is now apparently well. The remaining cases are so far free from recurrence.

Five cases have been operated upon during the last twelve months, and these are well at the present time.

It will be seen that so far there has only been one case in which local recurrence occurred; the question of internal deposits is one over which, as a rule, operation can have no control; almost always these deposits are present before operation. This series of cases includes all the cases of mammary cancer, which have come under my care, in which there has appeared any possibility of eradicating the disease. In two cases, the youth of the patients made the outlook serious. The youngest case was that of a girl, aged twenty, who had a carcinoma at the periphery of the right breast and was operated upon in July 1905; in the other case the patient, aged twenty-four, was admitted into the Grosvenor Hospital for hysterectomy for large fibroids, and a tumour discovered in the right breast was removed in June 1907, and proved carcinomatous. These two patients have remained well so far.

### Professor Halstead's Results.

Professor Halstead, who has done so much in the treatment of mammary cancer, has published a very full and careful summary of his results; and the figures in his paper form a powerful testimony to the success which follows thorough operating. Two hundred and ten cases are reported in which the result was traced: of these eighty-nine or 42.3 per cent. were well for three or more years, but fourteen of these developed recurrence later, leaving 75 or 35.7 per cent. of the cases which were accepted as cured.

This brings out one important point in regard to what is called the "three-year limit"; it is, of course, generally recognised that patients who remain free from recurrence for three years, cannot be regarded as cured; but these figures show that of those who remain well for three years 75 per cent. will probably be permanently cured.

One other very important fact is brought out in Professor Halstead's paper, and that is that the prognosis depends largely on the degree of glandular involvement; in sixty cases in which no axillary involvement could be demonstrated fifty-one passed the three-year limit and forty-five remained cured; whereas in 154 cases in which the glands were involved only thirty-eight passed the three-year limit, and thirty remained cured. In other words, when the axilla was apparently free, two cases in every three were cured; when the glands were involved three out of four died.

The prospects of success obviously vary greatly in different cases. The extent and situation of the disease, the character of the growth, and the general condition of the patient are factors which have powerful influence upon the prognosis. The results of operation in series of consecutive cases have been referred to above: these series include the good and the bad cases.

### Conclusions.

The obvious inference to be drawn from them is that when the growth is dealt with in an early stage and other conditions are favourable, permanent cure will probably follow operation; this statement may at first sight appear a bold one, but it is warranted by actual facts.

I have attempted to give some proof of the results of the radical operation for cancer of the breast. Many other series of cases might be quoted showing similar results; but I will only say that cases published by those surgeons who perform the radical operation show indisputably that the results obtained are infinitely better than those following the less complete operations. This fact seems a perfectly obvious one—it is exactly what one would expect. Yet there are many who vigorously oppose the full operation. Personally, I would urge most strongly that the complete operation be performed in every case in which there is any definite prospect of eradicating the disease, and I would most certainly not except the early cases. Some say that for the early and small growths, a less extensive operation is sufficient, but it is just in these early cases that the prospects of permanent cure are best, if a very thorough operation be done. It is important that the favourable results which follow thorough and early operations for mammary cancer should be widely known, especially amongst the public. For, after all, the greatest obstacle to progress is the fact that these tumours are so often concealed or watched; with each month of delay, the disease becomes more advanced, the prospects of cure diminish, and the operation becomes more serious; good results can only be obtained by early and thorough operations. If this is done, a large number of patients will be saved, who would otherwise die; and if we attempt to apply the same principle to the treatment of cancer in other parts of the body, we shall as a profession to some extent atone for our inability to find a cancer cure.

